# Solution of Some Types of Differential Equations: Operational Calculus and Inverse Differential Operators

**DOI:** 10.1155/2014/454865

**Published:** 2014-04-30

**Authors:** K. Zhukovsky

**Affiliations:** Faculty of Physics, M. V. Lomonosov Moscow State University, Leninskie Gory, Moscow 119899, Russia

## Abstract

We present a general method of operational nature to analyze and obtain solutions for a variety of equations of mathematical physics and related mathematical problems. We construct inverse differential operators and produce operational identities, involving inverse derivatives and families of generalised orthogonal polynomials, such as Hermite and Laguerre polynomial families. We develop the methodology of inverse and exponential operators, employing them for the study of partial differential equations. Advantages of the operational technique, combined with the use of integral transforms, generating functions with exponentials and their integrals, for solving a wide class of partial derivative equations, related to heat, wave, and transport problems, are demonstrated.

## 1. Introduction

Most of physical systems can be described by appropriate sets of differential equations, which are well suited as models for systems. Hence, understanding of differential equations and finding its solutions are of primary importance for pure mathematics as for physics. With rapidly developing computer methods for the solutions of equations, the question of understanding of the obtained solutions and their application to real physical situations remains opened for analytical study. There are few types of differential equations, allowing explicit and straightforward analytical solutions. It is common knowledge that expansion into series of Hermite, Laguerre, and other relevant polynomials [1] is useful when solving many physical problems (see, e.g., [2, 3]). Generalised forms of these polynomials exist with many variables and indices [4, 5]. In what follows, we develop an analytical method to obtain solutions for various types of partial differential equations on the base of operational identities, employing expansions in series of Hermite, Laguerre polynomials, and their modified forms [1, 6]. The key for building these solutions will be an operational approach and development of the formalism of inverse functions and inverse differential operators, already touched in [7, 8]. We will demonstrate in what follows that when used properly and combined, in particular, with integral transforms, such an approach leads to elegant analytical solutions with transparent physical meaning without particularly cumbersome calculations.

## 2. Inverse Derivative

For a common differential operator *D* = *d*/*dx* we can define an inverse derivative, such that upon the action on a function *f*(*x*) it gives another function *F*(*x*):

(1) $$\begin{array}{c} D^{-1}f\left(x\right)=F\left(x\right), \end{array}$$

whose derivative is *F*′(*x*) = *f*(*x*). Evidently, the inverse derivative *D* ^−1^ is executed by an integral operator being the inverse of differential operator, acting on *f*(*x*), and its general form is ∫*f*(*x*) = *F*(*x*) + *C*, where *C* is the constant of integration. The action of its *n*th order

(2) Dx−nf(x)=1(n−1)!∫0x(x−ξ)n−1f(ξ)dξ,(n∈N={1,2,3,…})

can be complemented with the definition of the action of zero order derivative as follows:

(3) $$\begin{array}{c} D_{x}^{0}f\left(x\right)=f\left(x\right), \end{array}$$

so that evidently

(4) $$\begin{array}{c} D_{x}^{-n}1=\frac{x^{n}}{n!},\;\left(n\in N_{0}=N\cup \left\{0\right\}\right). \end{array}$$

In what follows we will appeal to various modifications of the following equation:

(5) $$\begin{array}{c} {\left(\beta^{2}-D^{2}\right)}^{\nu }F\left(x\right)=f\left(x\right). \end{array}$$

Thus, it is important to construct the particular integral *F*(*x*) with the help of the following operational identity (see, e.g., [6]):

(6) q^−ν=1Γ(ν)∫0∞exp⁡(−q^t)tν−1dt,min⁡{Re(q),Re(ν)}>0.

For the operator $\hat{q}=\beta^{2}-D^{2}$ we have

(7) (β2−D2)−νf(x)  =1Γ(ν)∫0∞exp⁡(−β2t)tν−1exp⁡(tD2)f(x)dt.

We will also make explicit use of the generalized form of the Glaisher operational rule [9]; action of the operator $\hat{S}=\exp(tD_{x}^{2})$ on the function *f*(*x*) = exp⁡(−*x* ^2^) yields

(8) S^f(x)=exp⁡(y∂2∂x2)exp⁡(−x2)=11+4yexp⁡(−x21+4y).

Exponential operator mentioned above is closely related to Hermite orthogonal polynomials:

(9) $$\begin{array}{c} H_{n}\left(x,y\right)=n!\overset{\left[n/2\right]}{\underset{r=0}{\sum }}\;\frac{x^{n-2r}y^{r}}{\left(n-2r\right)!r!}, \end{array}$$

as demonstrated in [4, 5, 10] by operational relations:

(10) $$\begin{array}{c} H_{n}\left(x,y\right)=\exp\left(y\frac{\partial^{2}}{\partial x^{2}}\right)x^{n}. \end{array}$$

Moreover, the following generating function for Hermite polynomials exists:

(11) $$\begin{array}{c} \exp\left(xt+yt^{2}\right)=\overset{\infty }{\underset{n=0}{\sum }}\frac{t^{n}}{n!}H_{n}\left(x,y\right). \end{array}$$

Note also an easy to prove and useful relation [11]:

(12) $$\begin{array}{c} z^{n}H_{n}\left(x,y\right)=H_{n}\left(xz,yz^{2}\right). \end{array}$$

Laguerre polynomials of two variables [4]

(13) $$\begin{array}{c} L_{n}\left(x,y\right)=\exp\left(-y\frac{\partial }{\partial x}x\frac{\partial }{\partial x}\right)\frac{{\left(-x\right)}^{n}}{n!}=n!\overset{n}{\underset{r=0}{\sum }}\frac{{\left(-1\right)}^{r}y^{n-r}x^{r}}{\left(n-r\right)!{\left(r!\right)}^{2}} \end{array}$$

are related to the following operator ∂_*x*_ *x*∂_*x*_ [10]:

(14) DxL=∂∂xx∂∂x=∂∂Dx−1,

sometimes called Laguerre derivative _*L*_ *D*_*x*_. Note their noncommutative relation with the inverse derivative operator:

(15) [DxL,Dx−1]=−1,  ([A,B]=AB−BA).

They represent solutions of the following partial differential equation with proper initial conditions:

(16) $$\begin{array}{c} \partial_{y}L_{n}\left(x,y\right)=-\left(\partial_{x}x\partial_{x}\right)L_{n}\left(x,y\right), \end{array}$$

where

(17) $$\begin{array}{c} L_{n}\left(x,y\right)=n!\overset{n}{\underset{r=0}{\sum }}\frac{{\left(-1\right)}^{r}y^{n-r}x^{r}}{\left(n-r\right)!{\left(r!\right)}^{2}}, \end{array}$$

with proper initial conditions:

(18) $$\begin{array}{c} L_{n}\left(x,0\right)=\frac{{\left(-x\right)}^{n}}{n!}. \end{array}$$

In the following sections, we will investigate the possibilities to solve some partial differential equations, involving the differential operators studied above. Now we just note how the technique of inverse operator, applied for derivatives of various orders and their combinations and combined with integral transforms, allows for easy and straightforward solutions of various types of differential equations.

## 3. Diffusion Type, Heat Propagation Type Problems, and Inverse Derivatives 

Heat propagation and diffusion type problems play a key role in the theory of partial differential equations. Combination of exponential operator technique and inverse derivative together with the operational identities of the previous section is useful for the solution of a broad spectrum of partial differential equations, related to heat and diffusion processes. Some of them have been already studied by operational method (see, e.g., [10, 12]). Below we will focus on the generalities of the solution of the following problem:

(19) $$\begin{array}{c} \frac{\partial }{\partial y}F\left(x,y\right)=\left(\hat{P}+\hat{M}\right)\left\{F\left(x,y\right)\right\}, \end{array}$$

with initial conditions

(20) $$\begin{array}{c} F\left(x,0\right)=q\left(x\right). \end{array}$$

We will employ operational approach, combined with integral transforms and exponential operator technique. Formal solution for our generic formulation reads as follows:

(21) $$\begin{array}{c} F\left(x,y\right)=\exp\left(y\left(\hat{M}+\hat{P}\right)\right)q\left(x\right). \end{array}$$

We would like to underline that operators $\hat{P}$ and $\hat{M}$ may not commute, so, dependently on the value of their commutator, we will obtain different sequences of operators in (23), disentangling $\hat{P}$ and $\hat{M}$ [13, 14]. In the simplest case, when operators $\hat{M}$ and $\hat{P}$ are multiplication and differentiation operators, respectively, they can be easily disentangled in the exponent with account for

(22) $$\begin{array}{c} \left[\hat{P},\hat{M}\right]=1, \end{array}$$

which yields

(23) F(x,y)=exp⁡(y(M^+P^))q(x)=exp⁡(−y22)exp⁡(yP^)exp⁡(yM^)q(x).

Explicit expressions for the action of these operators on the initial condition function *g*(*x*) can be obtained by integral transforms, series expansions, and operational technique to be evaluated in every special case. This formulation, simple in its essence, nevertheless has wide application and allows us to frame either some of integrodifferential equations in this scheme. An elegant and interesting example is given by the following equation:

(24) $$\begin{array}{c} \frac{\partial }{\partial t}F\left(x,t\right)=-\frac{\partial }{\partial x}x\frac{\partial }{\partial x}F\left(x,t\right)+\overset{x}{\underset{0}{\int }}F\left(\xi ,t\right)d\xi ,\\ F\left(x,0\right)=g\left(x\right). \end{array}$$

Its formal solution reads

(25) $$\begin{array}{c} F\left(x,t\right)=\exp\left(\hat{A}+\hat{B}\right)g\left(x\right), \end{array}$$

where operators

(26) $$\begin{array}{c} \hat{A}=-t_{L}D_{x}=-t\frac{\partial }{\partial D_{x}^{-1}},\;\;\hat{B}=tD_{x}^{-1} \end{array}$$

do not commute:

(27) $$\begin{array}{c} \left[\hat{A},\hat{B}\right]=-t^{2}. \end{array}$$

Evidently, operators in the exponential disentangle:

(28) F(x,t)=exp⁡(A^+B^)q(x)=exp⁡(t22)exp⁡(tDx−1)exp⁡(−t∂∂Dx−1)g(x).

Thus, we have obtained the solution of the integrodifferential equation (24) as a sequence of exponential operators, transforming the initial condition *g*(*x*). Our further steps depend on the explicit form of this function. In the most general case of *g*(*x*) we may take advantage of the inverse derivative technique. First, consider the action of the operator exp⁡(−∂/(∂*D*_*x*_^−1^)) on *g*(*x*):

(29) $$\begin{array}{c} f\left(x,t\right)=\exp\left(-t\frac{\partial }{\partial D_{x}^{-1}}\right)g\left(x\right), \end{array}$$

where *D*_*x*_^−1^ is defined in (2). Equation (29) represents, in fact, the diffusion process and it is the solution of the following initial value problem:

(30) $$\begin{array}{c} \frac{\partial }{\partial t}f\left(x,t\right)=-\frac{\partial }{\partial x}x\frac{\partial }{\partial x}f\left(x,t\right),\;f\left(x,0\right)=g\left(x\right). \end{array}$$

Relevant studies were performed in [10, 12]. The initial condition function *f*(*x*, 0) = *g*(*x*) can be written as follows:

(31) $$\begin{array}{c} g\left(x\right)=\varphi \left(D_{x}^{-1}\right)1, \end{array}$$

and the image *φ*(*x*) is explicitly given by the following integral:

(32) $$\begin{array}{c} \varphi \left(x\right)=\overset{\infty }{\underset{0}{\int }}\exp\left(-\zeta \right)g\left(x\zeta \right)d\zeta , \end{array}$$

which is supposed to converge. Then the result of the Laguerre diffusion (29) appears in the form of the translation of the image function *φ*:

(33) $$\begin{array}{c} f\left(x,y\right)=\varphi \left(D_{x}^{-1}-t\right)1. \end{array}$$

Consequently, we have to apply the exponential operator exp⁡(*tD*_*x*_^−1^), which can be expanded in series:

(34) $$\begin{array}{c} F\left(x,t\right)=\exp\left(\frac{t^{2}}{2}\right)\overset{\infty }{\underset{n=0}{\sum }}\frac{t^{n}D_{x}^{-n}}{n!}f\left(x,t\right). \end{array}$$

The simplest example of the initial function *g*(*x*) = exp⁡(−*x*) demonstrates the technique sketched above, resulting in

(35) $$\begin{array}{c} \varphi \left(x\right)=\frac{1}{\left(1+x\right)}\;\text{when}\;\;\left|x\right|<1, \end{array}$$

and the Laguerre diffusion contribution (29) produces the following function:

(36) $$\begin{array}{c} f\left(x,t\right)=\frac{1}{1-t}\exp\left(-\frac{1}{1-t}x\right). \end{array}$$

The inverse derivative action on the exponent reads *D*_*x*_^−*n*^exp⁡(*αx*) = exp⁡(*αx*)/*α* ^*n*^ and eventually we obtain

(37) F(x,t)=exp⁡(t22)11−t∑n=0∞tnDx−nn!exp⁡(−11−tx)=11−texp⁡(−x1−t)exp⁡(−t(1−t)+t22).

In conclusion of the present chapter we consider the example of the solution of a heat propagation type equation by operational method, involving the inverse derivative operator and exponential operator technique. We recall that the common heat equation with initial condition problem

(38) $$\begin{array}{c} \frac{\partial }{\partial t}f\left(x,t\right)=\partial_{x}^{2}f\left(x,t\right),\;\;f\left(x,0\right)=g\left(x\right) \end{array}$$

can be solved by Gauss transforms:

(39) $$\begin{array}{c} f\left(x,t\right)=\frac{1}{2\sqrt{\pi t}}\overset{\infty }{\underset{-\infty }{\int }}\exp\left(-\frac{{\left(x-\xi \right)}^{2}}{4t}\right)g\left(\xi \right)d\xi . \end{array}$$

In complete analogy with the above statements, we can solve the heat-type equation with differential operator _*L*_ *D*_*x*_ (14) with, for example, the following initial condition:

(40) ∂∂tf(x,t)=Dx2Lf(x,t),  f(x,0)=g(x).

The Laguerre heat-type propagation problem (40) possesses the following solution (see the Appendix):

(41) f(x,t)=12πt∫−∞∞e−ξ2/4tC0H(−ξx2t,−x24t)φ(ξ)dξ.

## 4. Operational Approach and Other Types of Differential Equations

Operational approach to solution of partial differential equations, demonstrated on the examples of diffusion-like and heat-like equations with ∂_*x*_ *x*∂_*x*_ derivatives, can be further extended to other equation types. Consider the following example of a rather complicated differential equation:

(42) 1ρ∂∂tA(x,t)=x2∂2∂x2A(x,t)+λx∂∂xA(x,t)−μA(x,t), g(x)=A(x,0),

where *ρ*, *λ*, and *μ* are some arbitrary constant coefficients and function *g*(*x*) = *A*(*x*, *t* = 0) is the initial condition. By introducing operator $\overset{-}{D}=x\partial_{x}$ and distinguishing the perfect square, we rewrite (42):

(43) $$\begin{array}{c} \frac{1}{\rho }\frac{\partial }{\partial \;t}A\left(x,t\right)=\left({\left(\overset{-}{D}+\frac{\lambda }{2}\right)}^{2}-\varepsilon \right)A\left(x,t\right), \end{array}$$

where

(44) $$\begin{array}{c} \varepsilon =\mu +{\left(\frac{\lambda }{2}\right)}^{2}. \end{array}$$

Thus, the following exponential solution for (42) appears:

(45) $$\begin{array}{c} A\left(x,t\right)=\exp\left\{\rho t\left({\left(\overset{-}{D}+\frac{\lambda }{2}\right)}^{2}-\varepsilon \right)\right\}g\left(x\right). \end{array}$$

Now making use of the operational identity

(46) $$\begin{array}{c} \exp\left({\hat{p}}^{2}\right)=\frac{1}{\sqrt{\pi }}\overset{\infty }{\underset{-\infty }{\int }}\exp\left(-\xi^{2}+2\xi \hat{p}\right)d\xi \end{array}$$

and applying $\exp(a\overset{-}{D})$ according to

(47) $$\begin{array}{c} \exp\left(ax\partial_{x}\right)f\left(x\right)=f\left(e^{a}x\right), \end{array}$$

we obtain the following compact expression for *A*(*x*, *t*):

(48) A(x,t)=exp⁡(−ρεt)π×∫−∞∞exp⁡[−σ2+σαλ2ρ]g(xexp⁡⁡(σα))dσ,

where

(49) $$\begin{array}{c} \alpha =\alpha \left(t\right)=2\sqrt{\rho t}. \end{array}$$

Consider two simple examples of initial condition functions. The first one is

(50) $$\begin{array}{c} g\left(x\right)=x^{n}. \end{array}$$

Then we immediately obtain the solution of (42) as follows:

(51) $$\begin{array}{c} A\left(x,t\right)=x^{n}\exp\left\{\rho t\left(n^{2}+\lambda n-\mu \right)\right\}. \end{array}$$

The second example is given by the following initial condition function:

(52) $$\begin{array}{c} g\left(x\right)=\ln x. \end{array}$$

Trivial computations yield the following solution:

(53) $$\begin{array}{c} A\left(x,t\right)=\left(\ln x+\rho t\lambda \right)\exp\left(-\rho t\mu \right). \end{array}$$

Thus, operational technique, combined with integral transforms, operational identities, and extended forms of orthogonal polynomials, represents powerful tool for finding solutions of various classes of differential equations and initial value problems. Note that within the framework of inverse differential operators, developed and described above, the usage of the evolution operator method opens new possibilities, which we will elucidate in what follows.

Let us consider the following generalization of the heat equation:

(54) $$\begin{array}{c} \partial_{t}F\left(x,t\right)=\alpha \partial_{x}^{2}F\left(x,t\right)+\beta xF\left(x,t\right), \end{array}$$

with the initial condition:

(55) $$\begin{array}{c} F\left(x,0\right)=f\left(x\right). \end{array}$$

The evolution type equation (54) contains linear coordinate term in addition to the second order derivative. Its formal solution can be written via the evolution operator $\hat{U}$:

(56) $$\begin{array}{c} F\left(x,t\right)=\hat{U}f\left(x\right), \end{array}$$

where

(57) $$\begin{array}{c} \hat{U}=\exp\left(\hat{A}+\hat{B}\right),\;\hat{A}=\alpha t\partial_{x}^{2},\;\hat{B}=\beta tx. \end{array}$$

The exponential of the evolution operator $\hat{U}$ in (56) is the sum of two noncommuting operators and it can be written as the ordered product of two exponential operators. Indeed, the commutator of $\hat{A}$ and $\hat{B}$ has the following nonzero value:

(58) $$\begin{array}{c} \left[\hat{A},\hat{B}\right]=m{\hat{A}}^{1/2}=2\alpha \beta t^{2}\partial_{x},\;m=2\beta t^{3/2}\alpha^{1/2}. \end{array}$$

Then we can apply the disentanglement operational identity:

(59) $$\begin{array}{c} e^{\hat{A}+\hat{B}}=e^{(m^{2}/12)-(m/2){\hat{A}}^{1/2}+\hat{A}}e^{\hat{B}}, \end{array}$$

and the following chain rule:

(60) $$\begin{array}{c} e^{p\partial_{x}^{2}}e^{qx}g\left(x\right)=e^{pq^{2}}e^{qx}e^{2pq\partial_{x}}e^{p\partial_{x}^{2}}g\left(x\right), \end{array}$$

where *p*, *q* are constant parameters. With their help, we obtain the evolution operator $\hat{U}$ for (54):

(61) $$\begin{array}{c} \hat{U}=e^{\Phi (x,t;\beta )}\hat{\Theta }\hat{S}. \end{array}$$

Note that we have factorized two commuting operators: the operator of translation in space

(62) $$\begin{array}{c} \hat{\Theta }=e^{\alpha \beta t^{2}\partial_{x}} \end{array}$$

and operator

(63) $$\begin{array}{c} \hat{S}=e^{\alpha t\partial_{x}^{2}}, \end{array}$$

which is, in fact, operator $\hat{S}=\exp(tD_{x}^{2})$. The phase in $\hat{U}$ is written as follows:

(64) $$\begin{array}{c} \Phi \left(x,t;\alpha ,\beta \right)=\frac{1}{3}\alpha \beta^{2}t^{3}+\beta tx. \end{array}$$

The action of $\hat{U}$ on the initial condition function *f*(*x*) yields the following solution for our problem:

(65) $$\begin{array}{c} F\left(x,t\right)=e^{\Phi (x,t;\alpha ,\beta )}\hat{\Theta }\hat{S}f\left(x\right). \end{array}$$

Thus, we conclude from the form of (65) that the problem (54) with the initial condition (55) can be solved by the consequent application of commuting operators $\hat{\Theta }$ (62) and $\hat{S}$ (63) to *f*(*x*), apart from the factor *e* ^Φ(*x*,*t*;*α*,*β*)^. Now the explicit form of the solution (65) can be obtained by recalling that $\hat{\Theta }$ acts as a translation operator

(66) $$\begin{array}{c} e^{s\partial_{x}}g\left(x\right)=g\left(x+s\right) \end{array}$$

and that the action of $\hat{S}$ on the function *g*(*x*) yields the solution of the ordinary heat equation, through Gauss-Weierstrass transform ((A.1)). Accordingly, we denote

(67) $$\begin{array}{c} f\left(x,t\right)\equiv \hat{S}f\left(x\right)\equiv e^{\alpha t\partial_{x}^{2}}f\left(x\right), \end{array}$$

and we write

(68) $$\begin{array}{c} \hat{\Theta }f\left(x,t\right)=f\left(x+\alpha \beta t^{2},t\right). \end{array}$$

Thus, (54) with initial condition (55) has the following explicit solution:

(69) F(x,t) =eΦ(x,t;α,β)12παt∫−∞∞  e−(x+αβt2−ξ)2/4tαf(ξ)dξ,

provided that the integral converges. Summarizing the above outlined procedure, we conclude that a solution for (54) consists in finding a Gauss transformed function *f* with a shifted argument:

(70) $$\begin{array}{c} F\left(x,t\right)=e^{\Phi (x,t;\alpha ,\beta )}f\left(x+\alpha \beta t^{2},t\right). \end{array}$$

Moreover, this is a general observation for this type of equation, valid for any function *f*(*x*) (provided the integral converges). In other words, we have obtained the solution of Fokker plank equation as a consequent action of operator $\hat{S}$ of heat diffusion and operator $\hat{\Theta }$ of translation on the initial condition function. Note that *f*(*x*, *t*) is the solution of the heat equation, representing a natural propagation phenomenon.

The effect, produced by the translation operator $\hat{\Theta }$ and the operator $\hat{S}$, is best illustrated with the example of Gaussian *f*(*x*) = exp⁡(−*x* ^2^) evolution, when (69) becomes

(71) F(x,t)|f(x)=exp⁡(−x2)=exp⁡(Φ(x,t))1+4texp⁡(−(x+βt2)21+4t),t≥0.

The above result is exactly the generalization of Gleisher rule (8), considered earlier in the context of the heat equation. Thus, (71) is the solution of the ordinary heat equation with *β* = 0, when the initial function is Gaussian.

Another interesting example of solving (54) appears when the initial function *f*(*x*) allows the expansion in the following series:

(72) $$\begin{array}{c} f\left(x\right)=\sum_{k}c_{k}x^{k}e^{\gamma x}. \end{array}$$

In this case, we refer to the identity

(73) $$\begin{array}{c} \exp\left(yD_{x}^{2}\right)x^{k}e^{\gamma \;x}=e^{\left(\gamma \;x+\gamma^{2}y\right)}H_{k}\left(x+2\gamma y,y\right), \end{array}$$

which arises from

(74) $$\begin{array}{c} \exp\left(y\frac{\partial^{m}}{\partial x^{m}}\right)f\left(x\right)=f\left(x+my\frac{\partial^{m-1}}{\partial x^{m-1}}\right)1 \end{array}$$

with account for the generating function of Hermite polynomials (11). Then the action (67) of operator $\hat{S}$ on the initial function (72) produces

(75) $$\begin{array}{c} f\left(x,t\right)=\sum_{k}c_{k}f_{k}\left(x,t\right).\\ f_{k}\left(x,t\right)=e^{\gamma (x+\gamma a)}H_{k}\left(x+2\gamma a,a\right),\;a=\alpha t. \end{array}$$

In addition, the translation, operated by $\hat{\Theta }$, shifts the argument: *f*(*x*, *t*) → *f*(*x* + *ab*, *t*), *b* = *βt*. Thus, we obtain the solution in a form of Hermite polynomial as follows:

(76) $$\begin{array}{c} F\left(x,t\right)=\sum_{k}c_{k}e^{\Phi +\Phi_{1}}H_{k}\left(x+\frac{\alpha \gamma^{2}}{\beta }\left(2t\frac{\beta }{\gamma }+t^{2}\frac{\beta^{2}}{\gamma^{2}}\right),\alpha t\right), \end{array}$$

where Φ is defined by (64) and

(77) $$\begin{array}{c} \Phi_{1}=\gamma \left(x+\frac{\alpha \gamma^{2}}{\beta }\left(t\frac{\beta }{\gamma }+{\left(t\frac{\beta }{\gamma }\right)}^{2}\right)\right). \end{array}$$

It is now evident that, for a short time, when *t* ≪ *γ*/*β*, the solution will be spanning in space off the initial function, modulated by *H*_*k*_(*x*, *αt*) and exponentially depending on time:

(78) $$\begin{array}{c} {F(x,t)|}_{t\ll \gamma /\beta }\approx \sum_{k}c_{k}\exp\left(\alpha \gamma^{2}t+\gamma x\right)H_{k}\left(x,\alpha t\right),\\ {F(x,t)|}_{t\ll \gamma /\beta ,t\ll 1}\approx \sum_{k}c_{k}e^{\alpha \gamma^{2}t}e^{\gamma x}x^{k}. \end{array}$$

Note that for extended times *t* ≫ *γ*/*β* we have dominance of time variable: Φ ≫ Φ_1_, and the solution asymptotically behaves as exp⁡(*βt* *x*), while *x* plays minor role in *H*_*k*_(*x* + 2*γ* *αt* + *α* *βt* ^2^, *αt*).

The same operational technique as employed for the treatise of (54) can be easily adopted for the solution of the Schrödinger equation:

(79) $$\begin{array}{c} i\hbar \partial_{t}\Psi \left(x,t\right)=-\frac{\hbar^{2}}{2m}\partial_{x}^{2}\Psi \left(x,t\right)+Fx\Psi \left(x,t\right),\\ \Psi \left(x,0\right)=f\left(x\right), \end{array}$$

where *F* is constant (*F* has the dimension of force). Indeed, rescaling variables in (79), we obtain the form of equation, similar to (54):

(80) $$\begin{array}{c} i\partial_{\tau }\Psi \left(x,\tau \right)=-\partial_{x}^{2}\Psi \left(x,\tau \right)+bx\Psi \left(x,\tau \right), \end{array}$$

where

(81) $$\begin{array}{c} \tau =\frac{\hbar t}{2m},\;\;b=\frac{2Fm}{\hbar^{2}}. \end{array}$$

Following the operational methodology, developed for (54), we write the following solution of (80) in the form (56):

(82) $$\begin{array}{c} \Psi \left(x,\tau \right)=\hat{\overset{-}{U}}f\left(x\right), \end{array}$$

where

(83) $$\begin{array}{c} \hat{\overset{-}{U}}=\exp\left(i\tau \hat{H}\right),\;\hat{H}=\partial_{x}^{2}-bx. \end{array}$$

Then, on account of the substitution *t* → *iτ*, *β* → −*b*, operators

(84) $$\begin{array}{c} \hat{\overset{-}{\Theta }}=\exp\left(b\tau^{2}\partial_{x}\right),\;\;\hat{\overset{-}{\Theta }}f\left(x,\tau \right)=f\left(x+b\tau^{2},\tau \right), \end{array}$$

(85) $$\begin{array}{c} \hat{\overset{-}{S}}=\exp\left(i\tau \partial_{x}^{2}\right),\;\;\hat{\overset{-}{S}}f\left(x\right)=f\left(x,i\tau \right) \end{array}$$

arise. Thus, the solution of the Schrödinger equation is a result of consequent action of the operator $\hat{\overset{-}{S}}$ and further action of $\hat{\overset{-}{\Theta }}$ on the initial condition function:

(86) $$\begin{array}{c} \Psi \left(x,t\right)=\exp\left(-i\Phi \left(x,\tau ;b\right)\right)\hat{\overset{-}{\Theta }}\;\hat{\overset{-}{S}}f\left(x\right), \end{array}$$

where Φ is defined by (64). The integral form of the solution then is written as follows:

(87) Ψ(x,t)=exp⁡(−iΦ(x,τ;b))12iπτ×∫−∞∞exp⁡(−(x+bτ2−ξ)24iτ)f(ξ)dξ.

Again, as well as in (70), without any assumption on the nature of the initial condition function *f*(*x*) of the Schrödinger equation, its solution

(88) $$\begin{array}{c} \Psi \left(x,\tau \right)=\exp\left(-i\Phi \left(x,\tau ;b\right)\right)f\left(x+b\tau^{2},i\tau \right), \end{array}$$

where *f*(*x*, *t*) is given by (85) and is expressed in terms of the function of two variables, obtained by the consequent application of the heat propagation and translation operators (85) and (84) to*f*(*x*). So far we have demonstrated as Gauss-Weierstrass transform ((A.1)) describe the action of $\hat{\overset{-}{S}}$ on the initial probability amplitude and how the shift (84) finally yields the explicit form of the solution of Schrödinger equation. It means that the result of the action of evolution operator (83) on *f*(*x*) is the product of combined action of translation operator $\hat{\overset{-}{\Theta }}$ and heat propagation operator $\hat{\overset{-}{S}}$, representing the evolution operator of the free particle.

Now let us consider another Fokker-Plank type equation, that is, the following example:

(89) $$\begin{array}{c} \partial_{t}F\left(x,t\right)=\alpha \partial_{x}^{2}F\left(x,t\right)+\beta x\partial_{x}F\left(x,t\right), \end{array}$$

with initial condition (55). Proceeding along the above outlined scheme of the solution of (54), we write the solution of (89) in general form (56):

(90) $$\begin{array}{c} F\left(x,t\right)=\hat{U}f\left(x\right),\;\;\hat{U}=\exp\left(\hat{A}+\hat{B}\right), \end{array}$$

where operators $\hat{A}$ and $\hat{B}$ are defined as follows:

(91) $$\begin{array}{c} \hat{A}=t\alpha \partial_{x}^{2},\;\;\hat{B}=t\beta x\partial_{x}. \end{array}$$

Quantities $\hat{A}$ and $\hat{B}$ evidently do not commute:

(92) $$\begin{array}{c} \left[\hat{A},\hat{B}\right]=2\alpha \beta t^{2}\partial_{x}^{2}=m\hat{A},\;\;m=2t\beta . \end{array}$$

It allows disentanglement of the operators in the exponential according to the following rule:

(93) $$\begin{array}{c} \exp\left(\hat{A}+\hat{B}\right)=\exp\left(\frac{1-\exp\left(-m\right)}{m}\hat{A}\right)\exp\left(\hat{B}\right). \end{array}$$

Thus, the evolution operator $\hat{U}$ action on *f*(*x*), given below:

(94) U^f(x)=exp⁡(σ∂x2)exp⁡(βtx∂x)f(x)=exp⁡(σ∂x2)f(eβtx),

simply reduces to a Gauss transforms, where the parameter *σ* = *σ*(*t*) reads as follows:

(95) $$\begin{array}{c} \sigma \left(t\right)=\frac{\left(1-\exp\left(-2\beta t\right)\right)\alpha }{\beta }. \end{array}$$

Upon the trivial change of variables, we obtain

(96) $$\begin{array}{c} \hat{U}f\left(x\right)=\hat{S}f\left(y\right),\;y=x\exp\left(b\right),\;\;\hat{S}=\exp\left(\frac{\rho }{2}\partial_{y}^{2}\right), \end{array}$$

where

(97) $$\begin{array}{c} \rho \left(t\right)=\frac{\alpha }{\beta }\left(e^{2\beta t}-1\right) \end{array}$$

and, eventually, we end up with the following simple solution of (89):

(98) $$\begin{array}{c} F\left(x,t\right)=\frac{1}{\sqrt{2\pi \rho }}\overset{\infty }{\underset{-\infty }{\int }}e^{-{\left(\exp\left(\beta t\right)x-\xi \right)}^{2}/2\rho }f\left(\xi \right)d\xi . \end{array}$$

The same example of the initial Gaussian function *f*(*x*) = exp⁡(−*x* ^2^) as in the case of (54) yields (compare with (71))

(99) $$\begin{array}{c} {F(x,t)|}_{f(x)=\exp(-x^{2})}=\frac{1}{\sqrt{1+2\rho \left(t\right)}}\exp\left(-\frac{e^{2\beta t}x^{2}}{1+2\rho \left(t\right)}\right), \end{array}$$

where *ρ* is defined in (97). Note that we can meet the following modified form of Fokker-Plank type equation (89):

(100) $$\begin{array}{c} \partial_{t}F\left(x,t\right)=\alpha \partial_{x}^{2}F\left(x,t\right)+\beta \partial_{x}xF\left(x,t\right), \end{array}$$

in problems, related to propagation of electron beams in accelerators. Its solution arises from (99) immediately and differs from it just by a factor exp⁡(*βt*), as written below for a Gaussian *f*(*x*):

(101) F(x,t)|f(x)=exp⁡(−x2)=eβt1+2ρ(t)exp⁡(−e2βtx21+2ρ(t))=1η(t)exp⁡(−x2η(t)),η(t)=2αβ(1−e−2βt+β2αe−2βt).

However, differently from the solution of (54), where we had consequent transforms of the initial condition function *f*(*x*) by operators of translation and heat diffusion $\hat{S}$ (63) (see also [15]), here we have just the action of $\hat{S}$ alone with much more complicated dependence of the solution on time.

## 5. Conclusions

Operational method is fast and universal mathematical tool for obtaining solutions of differential equations. Combination of operational method, integral transforms, and theory of special functions together with orthogonal polynomials closely related to them provides a powerful analytical instrument for solving a wide spectrum of differential equations and relevant physical problems. The technique of inverse operator, applied for derivatives of various orders and combined with integral transforms, allows for easy and straightforward solutions of various types of differential equations. With operational approach, we developed the methodology of inverse differential operators and derived a number of operational identities with them. We have demonstrated that using the technique of inverse derivatives and inverse differential operators, combined with exponential operator, integral transforms, and special functions, we can make significant progress in solution of various mathematical problems and relevant physical applications, described by differential equations.

## Appendix

In complete analogy with the heat equation solution by Gauss-Weierstrass transform [16]:

(A.1) $$\begin{array}{c} \exp\left(b\partial_{x}^{2}\right)g\left(x\right)=\frac{1}{2\sqrt{\pi b}}\overset{\infty }{\underset{-\infty }{\int }}\exp\left(-\frac{{\left(x-\xi \right)}^{2}}{4b}\right)g\left(\xi \right)d\xi , \end{array}$$

accounting for noncommutative relation for operators of inverse derivative *D*_*x*_^−1^ and _*L*_ *D*_*x*_, defined through the operational relation (14) and accounting for (31) and (32), we write the solution of (40) in the following form:

(A.2) $$\begin{array}{c} f\left(x,t\right)=\frac{1}{2\sqrt{\pi t}}\overset{\infty }{\underset{-\infty }{\int }}\exp\left(-\frac{{\left({\hat{D}}_{x}^{-1}-\xi \right)}^{2}}{4t}\right)1\varphi \left(\xi \right)d\xi , \end{array}$$

where *φ* is the image (32) of the initial condition function *g*. The kernel of the integral in the above formula can be expanded into series of two-variable Hermite polynomials *H*_*n*_(*x*, *y*):

(A.3) exp⁡(−(D^x−1−ξ)24t)1=e−ξ2/4t∑n=0∞D^x−nn!Hn(ξ2t,−14t)=e−ξ2/4t∑n=0∞xn(n!)2Hn(ξ2t,−14t).

Taking into account formula (12) for *x* ^*n*^ *H*_*n*_(*ξ*/2*t*, −1/4*t*) in the operational identity above, which can be viewed as a generating function in terms of inverse derivative for *H*_*n*_(*x*, *y*), we obtain the following expansion for the kernel of the integral:

(A.4) $$\begin{array}{c} \exp\left(-\frac{\left({{\hat{D}}_{x}}^{-1}-\xi \right)}{4t}\right)1=e^{-\xi^{2}/4t}\overset{\infty }{\underset{n=0}{\sum }}\frac{1}{{\left(n!\right)}^{2}}H_{n}\left(\frac{x\xi }{2t},-\frac{x^{2}}{4t}\right), \end{array}$$

where the series of Hermite polynomials of two arguments *H*_*n*_(*x*, *y*) can be expressed in terms of Hermite-Bessel-Tricomi functions _*H*_ *C*_*n*_(*x*, *y*)—generalization of Bessel-Tricomi functions—and related to Bessel-Wright functions and to common Bessel functions [17]:

(A.5) CnH(x,y)=∑m=0∞Hm(−x,y)r!(n+m)!, n∈N0.

In particular, for *n* = 0, we immediately find our series:

(A.6) C0H(x,y)=∑m=0∞Hm(−x,y)(m!)2,

which obviously leads to the solution of the heat-type propagation problem (40) by the following appropriate Gauss transform:

(A.7) f(x,t)=12πt∫−∞∞e−ξ2/4tC0H(−ξx2t,−x24t)φ(ξ)dξ.
